# Urethane

**Published:** 1886-07

**Authors:** 


### Editorial.

---

### Urethane.

Some months since, the attention of the profession was directed to a new hypnotic and sedative called Urethane. It was greatly extoled, and claims made for it which seem to be fully sustained by repeated experiments and use.

The conclusions reached are that we have a powerful and yet very safe hypnotic. That it is very agreeable to take and does not interfere with any of the functions of the body. That upon its administration, calm sleep, free from dreams, follows in about ten to forty minutes. Some observers claim to have seen patients under the influence of the drug from twenty-four to forty-eight hours after one administration. All agree that it is not rapidly eliminated from the system. This is a fact we must bear in mind in its use, although it can be tolerated in large doses. It seems to be an established fact that the dose of this drug is from twenty to sixty grains, once or twice daily. "It is clear that the principal action of urethane is on the brain, without producing any marked irritation of the peripheral or sensory apparatus consequently, it is useless in the treatment of neuralgic pains, as well as in the pains of locomotor ataxia. But in other conditions where sleeplessness is the main symptom to be combated, urethane seems to possess the greatest advantages, since it is well borne by the patient; it produces absolutely no unpleasant symptoms, and the sleep which it provides is identical with normal physiological sleep. It would also appear that this remedy is particularly suitable for use in the treatment of diseases of children, where the need of a safe and sure hypnotic is greatly felt." We see from various reports that all agree it is well adapted to the treatment of insomnia, associated with heart and pulmonary troubles. It has a peculiar effect in insomnia, associated with this former class of diseases. There is a difference of opinion as to its action on the human system. Some observers claim that it acts simply on the cerebrum, and that it does not affect the respiratory or circulatory apparatus; while others say it acts on the spinal cord, and by its use the number ot heart-beats and respirations are reduced. This seems to be conclusively proven, although the former view is sustained by many prominent men, after careful research and investigation with the drug. Prof. Coze has shown that, physiologically, it is an antidote for strychnine.

At a recent meeting of the Medical Society in this city, an interesting paper was read on Urethane, the author reported many cases of sleeplessness treated by its use, with invariably good results. Those present related the successful use of the remedy in many cases of insomnia, associated with various diseases. The only failure reported was in a case of hysteria, where all remedies failed. In addition to the treatment of insomnia, we find it applied by Prof. Coze to the treatment of convulsions, especially tetanus. We should then give this new remedy a trial in epilepsy, chorea, tetanus and migraine; if it be found to be as valuable in this field as in that in which it already has a place, it will be a great boon to the profession.

